# Time trend of breast cancer mortality in BRAZILIAN men: 10-year data analysis from 2005 to 2015

**DOI:** 10.1186/s12885-018-5261-1

**Published:** 2019-01-07

**Authors:** Jean Henri Maselli-Schoueri, Fernando Alves Affonso-Kaufman, Claudia Vaz de Melo Sette, Francisco Winter dos Santos Figueiredo, Fernando Adami

**Affiliations:** 1 ABC Medical School – Santo Andre, Santo Andre, SP Brazil; 2 ABC Medical School, Oncology Department – Santo Andre, Santo Andre, SP Brazil; 3 ABC Medical School, Epidemiology and Data Analysis Laboratory, Santo Andre, SP Brazil

**Keywords:** Mortality, Time trend, Male breast Cancer, Brazil

## Abstract

**Background:**

Male Breast Cancer (MBC) is rare, which makes its understanding and treatment be extrapolated from what is known about the occurrence in women, with few epidemiological studies, with few epidemiological studies. Therefore, the aim of the present paper was to study breast cancer mortality in adult males in Brazil and its administrative regions between 2005 and 2015.

**Methods:**

Ecological study with data on MBC mortality in adults between 2005 and 2015. Data were obtained from the Mortality Information System of the Department of Informatics of SUS (the Unified Health System of the country). Descriptive statistics were used for MBC mortality and linear regression to analyze the relationship between mortality and the country’s administrative regions. Percentage Change (PC) and Annual Percentage Change (APC) were the trend measures used for MBC mortality for the period.

**Results:**

Between 2005 and 2015, there were 1521 deaths due to MBC in adults in Brazil. Regarding mortality by region, there was great oscillation in the rates of the country as a whole (PC = 113,87; β = 0,009 (IC95% 0,000 – 0,018); r^2^ = 0,381; *P* = 0,043). The highest increase in MBC mortality occurred in patients aged 80 years or older (PC = 161,04; β = 0,201 (IC95% 0,640 - 0,339); r^2^ = 0,550; *P* = 0,009) and there was significant increase in deaths for the 50–54-year age group (PC = 224,01; β = 0,135 (CI95% 0,052; 0,218); r^2^ = 0,601; P = 0,005).

**Conclusion:**

Mortality in adults due to MBC increased in Brazil during the study period with the highest percentage increase occurring for individuals aged 80 years or older.

**Electronic supplementary material:**

The online version of this article (10.1186/s12885-018-5261-1) contains supplementary material, which is available to authorized users.

## Background

Cancer is among the leading causes of death in the world [1]. In low-and middle-income countries, the disease accounts for about 10% of the 50 million annual deaths [2]. In Brazil, it is a public health problem [3] and data suggests it may be worsened by differences in income between populations of the country [4].

It is well-known that, in general, the sex of an individual implies differences in susceptibility to cancer [5], resulting in different rates of both incidence and mortality amongst women and men [6]. This comes to say that male breast cancer is rare [7] - with fewer than 6 cases per 100,000 people per year [8] -, which makes the understanding and treatment of the disease to be largely extrapolated from female breast cancer [9].

However, despite accounting for less than 1% of all breast cancer cases diagnosed worldwide [7], there is evidence that the incidence in men is increasing [10]. In terms of comparison, it is expected for 2017 that roughly 18, 5% of American men diagnosed with Male Breast Cancer (MBC) shall die due to the disease [11].

Having in mind the dynamic changes that be may occurring over time, the aim of the present study was to analyze the behavior of breast cancer mortality in adult males, in Brazil and its administrative regions, between 2005 and 2015.

## Methods

### Study design

This is an Ecological study conducted in 2018 with secondary data regarding breast cancer mortality in men between 2005 and 2015.

### Data source

Mortality data were collected from the Department of Informatics of the Unified Health System (DATASUS), maintained by the Brazilian Ministry of Health. The units of analysis were Brazil and its administrative regions (North, Northeast, South, Southeast and Midwest, as defined by the Brazilian Institute of Geography and Statistics) [12].

All data were collected by two independent researchers; a third researcher was responsible for correcting possible discrepancies. As for data reliability – and, thus, Internal Validity –, according to the most recent report, data coverage has been improving since the last decade, reaching 96.1% in 2011 [13]. Also, it should be borne in mind that, for years, the Brazilian mortality data, from a qualitative point of view, are accurate and reliable – similar to those of any country with a long tradition in the elaboration of these statistics [14]. Even so, we estimated the system’s coverage for the studied period by comparing the amount of deaths reported by the registry office to the amount found in the system for Neoplasms in men – as defined by Chapter II of the tenth revision of the International Classification of Diseases (ICD-10) [15]. (Additional file 1).

### Variables

The study was composed of all deaths recorded by the Mortality Information System (SIM) for breast cancer in adult men - 20 years or older - in Brazilian regions between the years of 2005 and 2015. The event under study - Breast Cancer - was defined according to ICD10 by code C50 [15].

In turn, the mortality rate was calculated by dividing the amount of deaths due to MBC by the corresponding male population of each region (for each year) and multiplying the result by 100,000. In order to be able to generalize our findings, the rates have then been standardized by the direct method, based on the age-group of the standard population - World Health Organization (WHO) [16]. It should be emphasized that in those cases, in which there was no number representing the amount of deaths in a certain region or time period, or even for a certain age group, the number “0” was considered for calculation purposes.

### Data analysis

To describe the mortality of breast cancer, descriptive statistics were performed. Linear regression was used to analyze the relationship between mortality and the administrative regions of Brazil throughout time.

The Percentage Change (PC) and the Annual Percentage Change (APC) are the two trend measures in this analysis. To calculate the PC, the initial value of the adjusted rate for breast cancer mortality is subtracted from its final amount, dividing the result by the same initial value of the rate and multiplying it by 100. For the APC, the angular coefficient or slope (β), derived from the linear regression, is used as shown by Fay et al., 2006 [17].

The confidence level was 95%. The statistical program used was Stata® (Stata Corp., College Station, EUA) 11.0.

## Results

Between 2005 and 2015, 1521 deaths were recorded for malignant male breast cancer in adults in Brazil. During this period, there was a large discrepancy in the distribution of death cases by administrative region of the country. While 749 deaths occurred in the Southeast region - almost half of the deaths for the period - only 5% of the cases could be found in the Northern Brazil (Table 1).

**Table 1:** Standardized mortality rates, absolute number and proportion of deaths, Percentage Change and Average Annual Percentage Change in Brazil, by administrative region, between 2005 and 2015

Administrative Region	Standardized Mortality^a^		Absolute Number of Deaths			Proportion of Deaths			PC^b^	APC^c^		
	2005	2015	2005	2015	2005–2015	2005	2015	2005–2015		β (CI 95%)	r^2^	P
North	0.2	0.29	4	11	79	6.45%	5.88%	5.19%	43.63	0.016	0.351	0.055
Northeat	0.06	0.3	6	44	371	9.68%	23.53%	24.39%	434	0.013	0.261	0.109
Southeast	0.19	0.37	37	102	749	59.68%	54.55%	49.24%	91.57	0.009	0.329	0.065
South	0.16	0.27	11	26	218	17.74%	13.90%	14.33%	68.35	0.007	0.131	0.274
Mid-west	0.12	0.1	4	4	104	6.45%	2.14%	6.84%	−14.94	−0.006	0.034	0.586
Brazil	0.15	0.32	62	187	1521	100%	100%	100%	113.87	0.009	0.396	0.038

^a^Standardized Mortality by 100,000 inhabitants ^b^PC: Percentage Change ^c^APC: Annual Percentage Change; β (CI 95%): angular coefficient or slope (95% Confidence Interval); r^2^: Model predictive capacity; *P*: linear regression

**Fig. 1:**
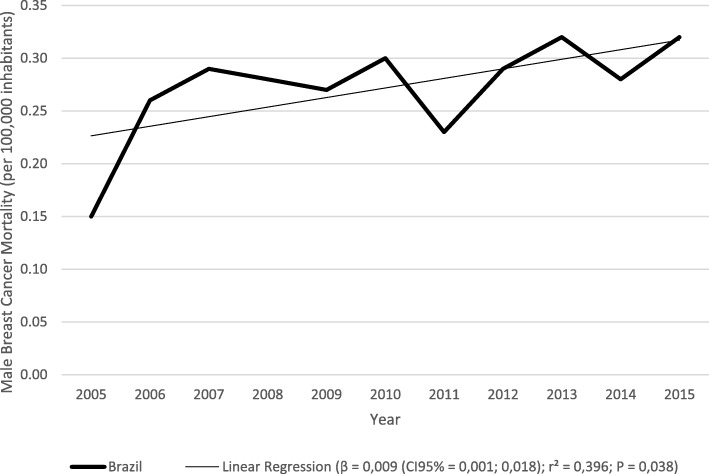
Time trend curve and linear regression of male breast cancer mortality in adults in Brazil between 2005 and 2015

Regarding the variation of the mortality rates for Brazil, there was a great oscillation during the period as a whole, although the annual variation being small (β = 0,009 (CI 95% 0,0001; 0,018); r^2^ = 0,396; *P* = 0,038) (Fig. 1).

However, in spite of the high values found in mortality rates variation throughout the period - represented by the high APC values - the analysis of each region separately did not present any statistically significant results (*P* > 0,05) (Table 1).

With regard to age-standardized mortality rates, on the other hand, it is possible to observe a predominance of deaths in the age groups above 50 years of age, with most advanced ages, such as those comprehended as 80 years or older, representing almost one-fifth of all deaths (PC = 161,04; β = 0,201 (CI 95% 0,063; 0,339); r^2^ = 0,548; *P* = 0,009). Still, the only other age group presenting statistically significant results was the one between 50 and 54 years of age, in which there was an increase in mortality rates in the studied period (PC = 224,01; β = 0,135 (CI95% 0,052; 0,218); r^2^ = 0,601; *P* = 0,005) (Table 2).

**Table 2:** Standardized Mortality, absolute number and proportion of deaths, Percentage Change and Annual Percentage Change in Brazil, by age group, between 2005 and 2015

Age Group	Standardized Mortality^a^		Absolute Number of Deaths			Proportion of Deaths			PC^b^	APC^c^		
	2005	2015	2005	2015	2005–2015	2005	2015	2005–2015		β (IC 95%)	r^2^	P
20 to 24 years	0.00	0.00	0.00	0.00	4.00	0.00%	0.00%	0.00%	–	−0.004	0.048	0.516
25 to 29 years	0.00	0.09	0.00	1.00	3.00	0.00%	0.01%	0.00%	–	0.002	0.017	0.705
30 to 34 years	0.00	0.17	0.00	2.00	17.00	0.00%	0.01%	0.01%	–	0.008	0.085	0.386
35 to 39 years	0.00	0.36	0.00	4.00	30.00	0.00%	0.02%	0.02%	–	0.013	0.044	0.538
40 to 44 years	0.44	0.38	4.00	4.00	56.00	0.06%	0.02%	0.04%	−12.18	−0.044	0.306	0.078
45 to 49 years	0.59	1.35	5.00	14.00	89.00	0.08%	0.07%	0.06%	129.47	0.014	0.024	0.653
50 to 54 years	0.64	2.09	5.00	22.00	132.00	0.08%	0.12%	0.09%	224.01	0.135	0.601	0.005
55 to 59 years	0.99	1.56	7.00	16.00	159.00	0.11%	0.09%	0.10%	57.32	0.052	0.161	0.221
60 to 64 years	1.10	1.83	7.00	18.00	206.00	0.11%	0.10%	0.14%	66.04	0.045	0.040	0.557
65 to 69 years	0.80	2.33	5.00	21.00	184.00	0.08%	0.11%	0.12%	190.33	0.039	0.031	0.606
70 to 74 years	0.84	1.85	5.00	15.00	163.00	0.08%	0.08%	0.11%	120.37	0.049	0.054	0.490
75 to 79 years	2.19	3.23	13.00	26.00	179.00	0.21%	0.14%	0.12%	47.82	0.080	0.140	0.258
80 years or older	2.09	5.45	11.00	44.00	299.00	0.18%	0.24%	0.20%	161.04	0.201	0.548	0.009
Total	0.15	0.32	62.00	187.00	1521.00	1.00%	1.00%	1.00%	113.87	0.009	0.396	0.038

^a^Standardized Mortality by 100,000 inhabitants ^b^PC: Percentage Change ^c^APC: Annual Percentage Change; β (CI 95%): angular coefficient or slope (95% Confidence Interval); r²: Model predictive capacity; *P*: linear regression

## Discussion

Only a few articles have studied MBC in Brazil, without demonstrating its patterns over time [18]. On the present paper, when analyzing the behavior of male breast cancer mortality in adults between 2005 and 2015 in Brazil, we found that:

i) There was a large oscillation of the age-standardized mortality rate in the country;
ii) There was a statistically significant increase in mortality rates for the age groups between 50 and 54 years and 80 or older.

To understand the results presented, some aspects must be taken into account. Regarding the age group included in the study, it should be considered that the literature shows a uni-modal distribution of the incidence of breast cancer cases in men, with a peak incidence at 71 years of age [19]. Even so, it had been decided to start collecting data on deaths of adult individuals - defined as those who present 20 years or more - in an attempt to acquire a representative sample of the male national panorama.

In addition, life expectancy for Brazilian men is 72.2 years of age, according to the official census of the Brazilian Institute of Geography and Statistics (IBGE) of 2016, which would make, a priori, the exclusive study of the age groups close to and above that mark something of little representativeness and relevance in national terms [20].

However, it is evident from the analysis of the results found that the largest sample size belongs to age groups comprised around and above 70 years, as described in the international literature [19, 21].

With regard to the oscillation in the mortality rates of Brazil and its regions, one can notice a great variation during the study period, with a small index of annual variation. Even though the regional analysis did not produce statistically significant results, it is interesting to note that data grouped throughout the country showed a significant increase, which leads us to question whether this would be a national trend, once technically the country’s analysis by region would work as a subgroup analysis and, therefore, more statistical power would be needed in order to find significance – which would not be the case for the country as a whole.

In this regard, the Medical literature shows that, in countries such as the United States and the United Kingdom, the incidence of this specific neoplasm has increased in recent years [22]. It should be taken into account that male breast cancer presents itself as equally or even less aggressive than the female counterpart [23], but its diagnosis in men often occurs in later stages of the disease [24, 25], which, in association with its increased incidence, corroborates the increase in mortality found in the present study.

In addition, there was a predominance of deaths in the age group comprised above 80 years, as shown by the proportion of deaths from 2000 to 2015 in Table 2, a finding that is partially in agreement with previously stated: men of any age can develop malignant neoplasm of breast – and the incidence of MBC tends to increase with age [26]. In this regard, the event becomes more common among the 60 to 70 year olds [27]. One possible explanation for this may lie simply in the way age groups have been divided (every 5 years). Thus, if we consider a single group ranging from 60 to 70 years, we shall find more than 25% of our sample, a fact consistent with the age described in the literature. However, it might also be the case that since we are working with small absolute numbers, there is a slightly high probability of both incidence and mortality matching, in the sense that where the incidence is higher, so was the mortality rate found.

Finally, however, it should be mentioned that stage of presentation and cancer subtype were some of the variables not considered for this paper due to the way data were collected, which alongside with income inequality from different national regions may have played a role in mortality rate changes as presented. For instance, it has been found that inequality of income was positively associated with an increase in female breast cancer mortality in Brazil [4]. Keeping this in mind, one could argue that there may be extrapolation from these results to MBC as well, which could lead to influences on its mortality rates.

All in all, there is the need to generate a greater focus on the disease in question. After all, as shown in the present study, the male population could benefit from a greater awareness, understanding, prevention and earlier diagnoses of malignant neoplasm of breast.

## Strengths and limitations

This is the first work, from the authors’ knowledge, to outline the evolution of male breast cancer mortality in adults over time in Brazil and its regions.

However, the extrapolation of the data presented here should be viewed with caution. Also, as mentioned earlier, MBC is a rare type of neoplasm, with few new cases and few absolute amount of deaths as well. Besides, considering that the present study is made of secondary data, there may be underreporting of the exact number of cases, which may have been aggravated if we take into account that, for calculation purposes, missing numbers were considered as null. Moreover, since the data collected were not individualized, their extrapolation to other units of analysis should not be performed.

## Conclusion

Mortality in adults due to male breast cancer increased in Brazil during the period studied, with the majority of deaths occurring in individuals aged 80 years or older.

## Additional file

Additional file 1: System’s coverage of deaths by Neoplasms from 2005 to 2013. (DOCX 13 kb)

## Abbreviations

APC: Annual Percentage Change
CI: Confidence Interval
DATASUS: Department of Informatics of the Unified Health System
IBGE: Brazilian Institute of Geography and Statistics
ICD10: 10th Revision of the International classification of diseases
MBC: Male Breast Cancer
PC: Percentage Change
SIM: Mortality Information System
WHO: World Health Organization
