# Supplementary material for: Time trend of breast cancer mortality in BRAZILIAN men: 10-year data analysis from 2005 to 2015
Source: BMC Cancer. 2019 Jan 7;19:23. doi: 10.1186/s12885-018-5261-1 (PMC6323830; doi:10.1186/s12885-018-5261-1)
Supplement: Supplementary file 1 — System’s coverage of deaths by Neoplasms from 2005 to 2013. (DOCX 13 kb) [file 12885_2018_5261_MOESM1_ESM.docx]

**Supplementary Material.** System’s coverage of deaths by Neoplasms from 2005-2013.

| Administrative Region | Year | | | | | | | | |
| --- | --- | --- | --- | --- | --- | --- | --- | --- | --- |
|  | 2005 | 2006 | 2007 | 2008 | 2009 | 2010 | 2011 | 2012 | 2013 |
| North | 60,72 | 63,03 | 65,29 | 69,91 | 68,37 | 71,80 | 72,96 | 74,76 | 75,68 |
| Northeast | 66,52 | 74,43 | 75,67 | 78,45 | 78,18 | 78,07 | 83,34 | 79,93 | 82,17 |
| Southeast | 85,75 | 88,30 | 87,58 | 88,48 | 88,08 | 90,29 | 91,07 | 89,59 | 90,86 |
| South | 91,73 | 92,26 | 94,58 | 92,70 | 94,59 | 93,72 | 97,36 | 93,03 | 96,63 |
| Mid-west | 86,51 | 85,71 | 86,50 | 90,70 | 86,45 | 90,39 | 91,80 | 90,62 | 89,69 |
| Brazil | 81,49 | 84,58 | 85,04 | 86,31 | 86,01 | 87,31 | 89,65 | 87,38 | 89,14 |
